# Femoral Cartilage Thickness in Knee Osteoarthritis Patients and Healthy Adults: An Ultrasound Measurement Comparison

**DOI:** 10.1155/2023/3942802

**Published:** 2023-02-17

**Authors:** Rita Vivera Pane, Rahayu Setiyaningsih, Gunawan Widodo, Aufar Zimamuz Zaman Al Hajiri, Juwita Raudlatul Salsabil

**Affiliations:** ^1^Department of Physical Medicine and Rehabilitation, Faculty of Medicine, Universitas Nahdlatul Ulama Surabaya, Surabaya 60237, Indonesia; ^2^Department of Physical Medicine and Rehabilitation, Hajj General Hospital of Surabaya, Surabaya 60116, Indonesia; ^3^Department of Internal Medicine, Hajj General Hospital of Surabaya, Surabaya 60116, Indonesia; ^4^Faculty of Medicine, Universitas Nahdlatul Ulama Surabaya, Surabaya 60237, Indonesia; ^5^Resident of Physical Medicine and Rehabilitation, Airlangga University, Surabaya 60115, Indonesia

## Abstract

**Background:**

Currently, conventional radiography is still widely used to diagnose knee osteoarthritis and assess the grade according to Kallgren and Lawrence's criteria. Ultrasound is a simple, inexpensive, noninvasive, and dynamic modality for evaluating femoral cartilage (FC) thickness. This study aims to measure the FC thickness in knee osteoarthritis (OA) patients and compare it to healthy adults using ultrasound assessment.

**Methods:**

An observational study with a cross-sectional design was conducted at the Department of Physical Medicine and Rehabilitation of Hajj General Hospital, Surabaya, Indonesia, from May to July 2022. Participants radiologically diagnosed with OA were included in the study and assigned to the OA group. Meanwhile, healthy adults without knee symptoms were included in the control group. FC thickness was measured using ultrasound scans at three sites: medial condyle (MC), intercondylar (IC), and lateral condyle (LC) on both sides of the knee.

**Results:**

The mean age in the OA and control groups was 61.03 ± 8.6 and 33.93 ± 14.7 years, respectively. Most participants in both groups were female. The OA group exhibited a thinner FC (1.49–1.63 mm) than the control group (1.68–1.87 mm). There was a significant difference in the mean of the right and left MC in both groups (*p* < 0.05) but no significant difference in the IC and LC.

**Conclusion:**

OA patients exhibited a thinner FC than healthy adults in the control group. There was a significant difference in the mean thickness of the MC between groups.

## 1. Introduction

Knee osteoarthritis (OA) is a common and frequent cause of knee symptoms. Knee OA increases with age, and in people above 65 years of age, OA is reported to occur in 29.1% of men and 41.5% of women. Since 2018, the prevalence of OA in Indonesia has been 8.5% and 6.1% in women and men, respectively. Meanwhile, middle-aged individuals with no knee symptoms often exhibit a partial thickness defect in the medial (61%) rather than the lateral site (43%), associated with cartilage loss with aging. However, the association between the full thickness of cartilage defects and the risk of developing knee OA in older age is unclear. A full-thickness cartilage defect leads to inflammation and is a common source of knee pain [1–3].

Osteoarthritis primarily occurs in the joints but often affects bone, cartilage, synovial, meniscus, ligaments, muscles, nerves, bursa, and fat pads around. Conventional radiography is the current primary modality for diagnosing knee OA and evaluating the severity grade. However, conventional radiology has its limitations. Recent studies have used ultrasound to evaluate patients with knee symptoms. It is a simple, inexpensive, flexible, and dynamic modality with no radiation exposure, making it safer for patients. One of the advantages of ultrasound lies in its ability to evaluate soft tissue structure and articular cartilage integrity and thickness. Femoral cartilage (FC) on ultrasound appears as a homogeneous anechoic structure. In addition, ultrasound can also detect early changes in the synovial joint, meniscus, and osteochondral tissue, which are signs of knee OA abnormalities [4–8].

The full-thickness cartilage defects are still unclear regarding the mechanism of OA development. Previous studies reported that ultrasound-measured FC thickness correlated with macroscopically assessed cartilage thickness. As measured by ultrasound, the thickness of the articular cartilage has the same result as the histological measurements [1, 4, 9].

This study aims to determine the ultrasound measurement of femoral cartilage thickness among knee osteoarthritis patients and compare them to healthy adults.

## 2. Materials and Methods

### 2.1. Study Participants

This cross-sectional observational study was conducted at the Physical Medicine and Rehabilitation Department of Hajj General Hospital East Java, Surabaya, Indonesia, from May to July 2022. The study was divided into two groups of participants including OA and control groups. Patients registered with hospital medical records diagnosed with knee OA based on clinical and radiographic features with Kallgren and Lawrence grade I–IV criteria were included in the OA group. Meanwhile, the control group recruited hospital employees, medical students, and patient families waiting around the Physical Medicine and Rehabilitation Department with no complaints of knee pain. All participants who met the inclusion criteria obtained informed consent to be involved in this study. Participants with autoimmune disease, a history of trauma, and surgery around the knee were excluded from this study.

### 2.2. Measurement

**Figure 1:**
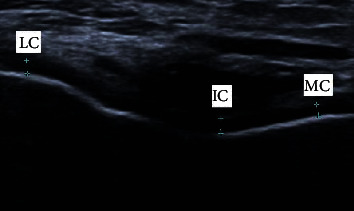
Transversal ultrasound suprapatellar scan of the knee joint in right femoral cartilage; lateral condyle (LC), intercondylar (IC), and medial condyle (MC).

The FC measurement was performed using a 2D ultrasound device. This study used Canon Xario 100 with a 18L7 linear transducer 7.2–14 MHz. Focus and depth setting was customized for each patient according to their condition. The scanning was performed by a single examiner, i.e., a physiatrist with five years of experience and certified training in ultrasound for interventional pain management. The FC thickness was measured with each patient in a supine position and full knee flexion. The US scanning was performed transversally in the intercondylar notch of the distal femur. The thickness measurements of the medial condyle (MC), intercondylar (IC), and lateral condyle (LC) were performed on both sides, presented in millimeters (see Figure 1).

### 2.3. Statistical Analysis

This study used Microsoft Excel to collect all research data and IBM SPSS version 26 to analyze statistical data. Demographic data such as age, gender, weight, height, and BMI were analyzed descriptively. The correlation between variables was analyzed using Pearson Chi-square. FC thickness data are presented as mean ± SD. Normality and homogeneity tests showed that the data were not normally distributed and homogeneous, respectively. Therefore, a nonparametric statistical analysis was performed. The mean comparison of each group was analyzed using the Mann–Whitney test with a significance value of *p* < 0.05.

### 2.4. Ethical Permission

This study was performed after receiving ethical permission from the Ethical Committee of Hajj General Hospital of Surabaya, with the letter number 073/07/KOM.ETIK/2022. All patients were given information on the measurement procedure and stated their consent prior to measurements.

## 3. Results and Discussion

### 3.1. Result

A total of 60 participants were involved in this study. Participants were divided into two groups: OA and control groups, and each group comprised 30 participants. Demographic data such as age, gender, weight, height, and BMI are analyzed in Table 1. The correlation between variables showed a significant correlation with age (*p* < 0.05) but was not significant in another variable.

The FC thickness in both groups was recorded and stated as mean in millimeters (see Table 2). The between-group analysis showed a significant mean difference in MC on both sides (*p* < 0.05) but no significant mean difference in both IC and LC.

### 3.2. Discussion

In this study, the OA group showed a thinner FC mean than the control group at the six measurement sites (see Table 2). This finding is in line with a previous study conducted in Saudi Arabia, which also reported a thinner FC in patients with knee OA than in healthy subjects. The OA group showed a thinner FC in the six measurement locations [10]. When compared to FC thickness among healthy adults, several studies showed thicker mean FC [11, 12].

These findings showed a significant difference in measurements on the right and left MC sides between the OA and control groups. In line with several studies showing a significant correlation in MC side measurements between knee OA and non-OA groups, the MC side was thinner than the LC and IC sides [13–15]. Cartilage defects in knee OA patients often occur on the MC side, especially in the tibiofemoral compartment, which lead to knee pain. The cartilage thickness in this area is sensitive to changes associated with the development of OA and is radiographically related to the width of the joint space. However, another study showed that there was thickening of the nonload bearing area on the posterior MC side, which was absent on the LC and IC sides. The thickening of the posterior area of the MC depends on the severity of the OA. Currently still unclear why the posterior MC is thicker in the knee with severe OA [2, 16–18].

The measurement of the MC side showed significant between-group differences in mean thickness of both RMC and LMC. This was caused by the ground reaction force passing straight medially to the knee joint, thus creating a varus torsion in the knee with each step of walking. The moment arm is elongated between the anterior-posterior axis and the ground reaction force. The medial articular surface of the joint receives a greater compression force. The MC and LC are asymmetrical in morphology, wherein the LC is flatter, longer in anteroposterior, and higher than the MC. Moreover, the articular surface of the MC is oval, while the LC is circular. This difference leads to an important effect on knee joint motion [19, 20]. The IC measurement, both RIC and LIC sides, tend to be the thickest when compared to the MC and LC sides. But there was no significant difference between the OA and control groups. In line with previous studies in healthy people without knee OA, the IC side was thickest compared to the MC and LC sides [21].

Some participants in the OA group showed extreme thickness differences, where they were thicker than the control group. Another study reports a higher mean of MC thickness in OA knees than in healthy knees. It may be associated with swollen cartilage, as it commonly occurs in the early stages of knee OA. Previous studies using animal models suggested that the cartilage in the thickened area is possibly hypertrophic, which is the effect of the anabolic processes of knee OA. The present finding is consistent with previous studies reporting that thicker calcified cartilage (CC) is present in the early stages of knee OA. In areas without loss of articular cartilage (AC) thickness, studies found that bone formation via endochondral ossification is an early knee OA. Regarding the LC, there was no statistically significant difference in the mean cartilage thickness between the knee OA and the healthy one [14, 22].

The limitation of this study lies in its use of a single examiner. It should be noted that ultrasound examiners may have different handling and interpretation, depending on their experience.

## 4. Conclusions

Femoral cartilage in the OA group was thinner than that of the control group. This study showed a significant mean difference in RMC and LMC thickness between OA and control groups but no significant difference in RIC, LIC, RLC, and LLC. Ultrasound is a simple, noninvasive, and radiation-free modality for evaluating the FC thickness.

## Figures and Tables

**Table 1:** Demographic data between osteoarthritis and control groups.

	Osteoarthritis (*n* = 30)	Control (*n* = 30)	*p*
Age, years (mean ± SD)	61.03 ± 8.6	33.93 ± 14.7	0.02
Male, *n* (%)	5 (8.4%)	9 (15.0%)	0.12
Female, *n* (%)	25 (41.6%)	21 (35.0%)	
Weight, kg (mean ± SD)	62.13 ± 9.6	60.37 ± 8.5	0.55
Height, cm (mean ± SD)	157.70 ± 7.0	161.80 ± 8.2	0.15
BMI (mean ± SD)	24.96 ± 3.4	23.12 ± 3.3	0.33

**Table 2:** Ultrasound measurement of femoral cartilage thickness between osteoarthritis and control groups, in millimeters.

Mean ± SD	Osteoarthritis (*n* = 30)	Control (*n* = 30)	*p* ^*∗*^
RMC	1.49 ± 0.54	1.81 ± 0.33	0.01
RIC	1.62 ± 0.75	1.87 ± 0.37	0.13
RLC	1.63 ± 0.50	1.82 ± 0.32	0.25
LMC	1.59 ± 0.59	1.85 ± 0.28	0.04
LIC	1.60 ± 0.64	1.83 ± 0.34	0.12
LLC	1.52 ± 0.53	1.68 ± 0.29	0.36

RMC: right medial condyle; RIC: right intercondylar; RLC: right lateral condyle; LMC: left medial condyle; LIC: left intercondylar; LLC: left lateral condyle. ^*∗*^Mann–Whitney test.

## Data Availability

The data used to support the findings of this study are available from the corresponding author upon reasonable request.
